# Temperature Controlled Mechanical Reinforcement of Polyacrylate Films Containing Nematic Liquid Crystals

**DOI:** 10.3390/polym14225024

**Published:** 2022-11-19

**Authors:** Latifa Zair, Abdelkader Berrayah, Khadidja Arabeche, Zohra Bouberka, Andreas Best, Kaloian Koynov, Ulrich Maschke

**Affiliations:** 1 Laboratoire de Recherche sur les Macromolécules (LRM), Faculté des Sciences, Université AbouBekr Belkaïd de Tlemcen (UABB), BP 119, Tlemcen 13000, Algeria; 2 Unité Matériaux et Transformations (UMET), UMR 8207, University Lille, CNRS, INRAE, Centrale Lille, 59000 Lille, France; 3 Laboratoire Physico-Chimie des Matériaux-Catalyse et Environnement (LPCM-CE), Université des Sciences et de la Technologie d’Oran Mohamed Boudiaf (USTOMB), BP 1505, El M’naouer, 31000 Oran, Algeria; 4 Max-Planck Institut für Polymerforschung, Postfach 3148, 55121 Mainz, Germany

**Keywords:** polyacrylates, liquid crystals, electron beam curing, UV-visible irradiation, dynamical mechanical analysis, elastic modulus, glass transition

## Abstract

This investigation reports on the thermomechanical properties of Poly-tripropyleneglycoldiacrylate (Poly-TPGDA)/liquid crystal (LC) blends, developed via free radical polymerization processes, which are induced by Electron Beam (EB) and Ultraviolet (UV) radiation. The EB-cured Poly-TPGDA network exhibits a higher glass transition temperature (*T*_g_), a higher tensile storage, and Young moduli than the corresponding UV-cured sample, indicating a lower elasticity and a shorter distance between the two adjacent crosslinking points. Above *T*_g_ of Poly-TPGDA/LC blends, the LC behaves as a plasticizing agent, whereas, for EB-cured networks, at temperatures below *T*_g_, the LC shows a strong temperature dependence on the storage tensile modulus: the LC reinforces the polymer due to the presence of nano-sized phase separated glassy LC domains, confirmed by electron microscopy observations. In the case of the UV-cured TPGDA/LC system, the plasticizing effect of the LC remains dominant in both the whole composition and the temperature ranges explored. The rubber elasticity and *T*_g_ of Poly-TPGDA/LC films were investigated using mechanical measurements.

## 1. Introduction

Radiation curing of reactive functional monomers and oligomers represents a widely used technique for academic purposes and for industrial applications, such as thin film coatings, adhesives, and paintings [1,2,3,4,5,6,7], especially since the desired reactions are generally ultrafast, they can be carried out at an ambient temperature, and do not need the use of solvents or catalysts. A broad choice of reactive initial polymer precursors is commercially available, which can be adapted for particular applications by choosing the correct basic compositions. Several parameters, such as number and volume densities of functional groups, molar weights, chemical structures, viscosities, and the processing temperature can be adjusted to obtain the required polymeric material.

Acrylates are cheap and versatile monomeric materials that can be easily transformed by radiation polymerization and crosslinking reactions to obtain stable material properties [8]. During exposure to radiation, the initial reactive blends will be transformed to stable three-dimensional chemically cross-linked polymer networks, which can be characterized, for example, by their glass transition temperatures and their average distance between two neighboring cross-linking points.

The choice of curing conditions, such as radiation dose and dose rate, strongly influences the kinetics of polymerization/cross-linking processes, and thus has an impact on the formation of the polymer network. Ultraviolet (UV)—visible-induced radical polymerization processes generally require the presence of a photoinitiator absorbing in the emission spectrum of the employed radiation source. The electron beam (EB) technique has the advantage of enabling the curing process to be carried out in the absence of any initiating species and is not sensitive to UV-absorbent materials, for example dyes. This technique has already been applied to elaborate polymer films and Polymer Dispersed Liquid Crystals (PDLC) materials [9,10,11,12,13]. Starting from a homogeneous mixture with a low molecular weight LC, and the reactive monomer(s), radiation induced polymerization/crosslinking processes lead to a decrease in the miscibility between the LC and the growing polymer network, with the mixture finally separating into two phases. Depending on the LC concentration range, PDLC materials can be obtained, consisting of micron sized LC domains distributed randomly in the polymer matrix. These materials are still subject to intensive studies because of the fundamental interests and their potential applications, including intelligent windows, information displays, optical shutters, and holographic devices [14,15,16,17,18,19]. For example, normal mode PDLC films can be switched from being optically opaque to a transparent state with the application of an electrical field.

In this work, the influence of adding an LC to a difunctional acrylic monomer, prior to its irradiation, was investigated in detail, with particular attention to the concept of adding small filler particles to a polymer matrix. Research also focuses on nano- and micro-composite polymers in order to achieve a strengthening of the virgin polymer [20,21,22,23,24,25,26,27,28,29,30], with relation to specific chemical and physical properties of the filler particles. To the best of our knowledge, only a few reports are available on studies for reinforced polymers developed by radiation curing of acrylic monomer/filler blends [29,30,31,32,33,34].

The experimental mechanical data from selected cured polymer/LC systems will be presented and analyzed in order to understand the effect of UV/EB radiation on both of the polymer and the LC properties. The LC is expected to either act as a plasticizer, or to reinforce the polymer, depending on the composition and the temperature.

## 2. Materials and Methods

### 2.1. Materials

The LC E7 was purchased from Synthon GmbH (Wolfen, Germany). It is an eutectic mixture exhibiting a nematic-isotropic transition temperature of *T*_NI_ = 60 °C, a glass transition of *T*_g_ (LC) = −62 °C, and a volumetric density of 1.03 g/L at room temperature. E7 is composed of 51 weight percent (wt.%) 4-cyano-4′-n-pentyl-biphenyl (5CB, molecular weight 249.36 g/mol), 25 wt.% 4-cyano-4′-n-heptyl-biphenyl (7CB, molecular weight 277.41 g/mol), 16 wt.% 4-cyano-4′-n-octyloxy-biphenyl (8OCB, molecular weight 307.44 g/mol), and 8 wt.% 4-cyano-4″-n-pentyl-p-terphenyl (5CT, molecular weight 325.46 g/mol), providing the wide temperature range of a nematic phase (≈120 °C) [35]. E7 can thus be characterized by a theoretical molecular weight of 271.75 g/mol. Tripropyleneglycoldiacrylate (TPGDA, molecular weight 300 g/mol) as a difunctional monomer, was purchased from Merck (Sigma Aldrich, Saint-Quentin-Fallavier, France). All materials were used without further purification.

### 2.2. Preparation of Monomer/LC Blends

The TPGDA/E7 blends containing x wt.% of TPGDA and (100-x) wt.% E7 were homogenized at an ambient temperature. The samples were applied uniformly on glass plates and irradiated at room temperature in a nitrogen atmosphere, either by exposure to EB irradiation or to UV light. 2 wt.% (with respect to the monomer) of Lucirin TPO (BASF) as a photoinitiator, was added to the initial mixture in the latter case. Film thicknesses of 100 µm were prepared to allow for complete and homogeneous penetration of EB and UV irradiation.

### 2.3. Electron Beam Curing

The EB generator is an Electrocurtain Model CB 150 (Energy Sciences Inc., Wilmington, MA, USA), delivering a voltage of 175 kV. The samples were prepared according to the above procedure and placed on a tray, which went under the irradiation source using a conveyor belt. Samples were exposed to a dose of 105 kGy, which was achieved using a constant conveyor speed of 0.19 m/s and a beam current of 7 mA. For each composition, several samples were prepared to ensure the validity of the results.

### 2.4. Ultra Violet Curing

The UV light source used was a Minicure Model MC4-300 (Primarc UV Technology, Slough, Great Britain) equipped with a medium pressure mercury arc lamp rated 80 W/cm. The samples were placed on a conveyor belt and exposed to UV light, applying a dose of 150 mJ/cm^2^.

### 2.5. Mechanical Measurements

Static properties were analyzed using a mechanical testing machine, Instron 6022. Rectangular samples were cut from irradiated films and the effective sample dimensions were roughly 15 × 4 × 0.1 mm^3^. Measurements were performed at 20 °C at a constant rate of 1 mm/min. The stress vs. draw ratio *λ,* was recorded where *λ* is the ratio of the final length *l* to the initial length *l*_0_, prior to the application of stress. Five to eight independent measurements were made and the results represent average values. Young moduli were determined from the slope of the stress/draw ratio curves at a zero strain.

Dynamic measurements were performed by means of a Rheometrics RMS 800 mechanic spectrometer. Rectangular samples with effective dimensions of 20 × 4 × 0.1 mm^3^ were cut from the prepared films. Uniaxial tensile deformation was applied under the condition of a controlled deformation amplitude, which was changed with temperature between Δ*γ* = 0.0001 at low and Δ*γ* = 0.05 at high temperatures, remaining in the range of a linear viscoelastic response. A special set-up designed for the investigated films was used and the experiments were performed under a dry nitrogen atmosphere.

Curves representing storage and loss moduli (*E*’ and *E”*) were measured at a constant deformation frequency of 10 rad/s and a heating rate of 2 °C/min, starting from *T* = −100 °C up to the temperature where a rubbery state plateau modulus was detected. Values of *tan δ* were deduced from the ratio of loss to storage moduli.

### 2.6. Scanning Electron Microscopy

Samples for Scanning Electron Microscope (SEM) studies were prepared in the same way as described above. After irradiation exposure was finished, the samples were immersed in tetrahydrofuran for several seconds, in order to extract the LC. The samples were then coated by a thin platinum layer and characterized by a SEM (Philips XL-30 field emission gun). This sample preparation technique leads to the appearance of dark holes that were once filled with LC. The magnification used in our experiments was about 5000× using an acceleration voltage of 10 kV.

## 3. Results and Discussion

### 3.1. Development of Crosslinked Polymer/LC Films

The effects of both radiation dose and dose rate on the preparation of UV- and EB-cured polymer and polymer/LC films were investigated by Bouchakour et al. [12,13] on TPGDA/E7 and other similar acrylic systems. Polymerization and crosslinking reactions of monomer/LC mixtures may introduce phase separation processes that are likely to govern sample morphologies of the obtained polymer/LC films. Phase diagrams of monomeric TPGDA/E7, as well as from the corresponding UV- and EB-cured systems, were determined using several analytical techniques, such as light microscopy, differential scanning calorimetry, and light scattering, showing a one-phase region for both cured systems below 30 wt.% of LC, and a broad biphasic nematic + isotropic region above this concentration [36,37,38].

The curing conditions for each radiation method (UV and EB) were chosen to give a fair comparison between the corresponding mechanical results presented in this report. The UV irradiation equipment (Minicure) was adopted to be able to attain a fast curing speed comparable to that used for EB curing, i.e., the maximum exposure time did not exceed 3 s. The dose values were fixed to reach complete conversion of the monomer TPGDA into a chemically crosslinked polymer network. Indeed, high acrylic double-bond conversions should be reached to minimize the undesired effects of unreacted or partially reacted monomer molecules. In particular, the decrease in the FTIR-bands associated with the carbon-carbon double bonds of the acrylate groups were followed (C=C deformation band at 810 cm^−1^, and another C=C valence vibration around 1638 cm^−1^), as a function of a radiation dose. It was found that increasing the radiation doses lead to an increase in the acrylic double bond conversion [13]. The LC E7 acts as a solvent and the polymerization reaction was governed by the diffusion of the initiator radicals in this solvent towards the acrylic double bonds. When the phase separation began, the E7 segregated in small domains and the reaction was then enhanced towards higher conversions. Polymerization/crosslinking reactions involving E7 reached a 100% monomer conversion due to a certain mobility of the reactive species in this solvent. On the other hand, in the absence of E7, TPGDA conversion was limited to around 90% due to the relationship with the strongly reduced mobility of the radicals in the glassy state of the irradiated pure polymer matrix.

As a result, a complete monomer conversion was achieved at radiation doses of 105 kGy for the EB-cured and 150 mJ/cm^2^ for the UV-cured TPGDA/E7 systems, this then enabled the two radiation methods to be compared at their best performance.

### 3.2. Static Mechanical Measurements

**Figure 1:**
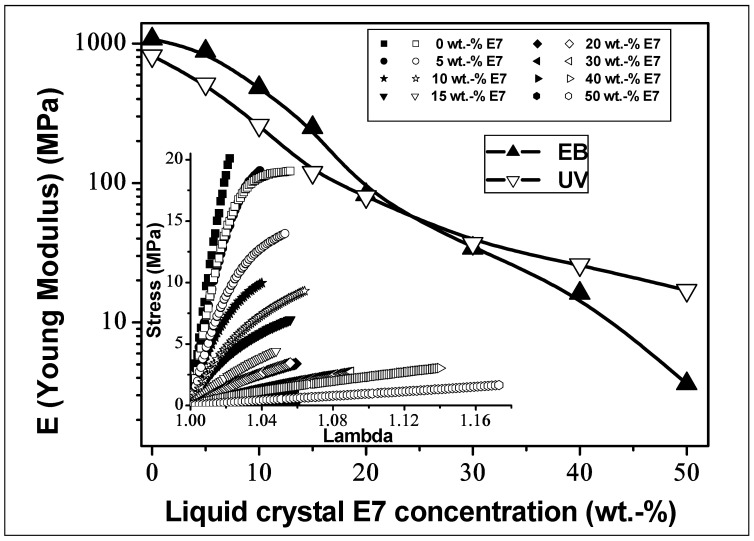
Static mechanical analysis: Dependence of Young modulus on LC concentration for both EB- and UV-cured TPGDA/E7 systems. The insert shows stress as a function of draw ratio *λ* for the same samples.

Figure 1 represents the evolution of Young moduli as a function of the LC content for both EB- and UV-cured TPGDA/E7 blends in the range from 0 to 50 wt.% of E7. Starting from *E*_EB_ = 1080 MPa and *E*_UV_ = 820 MPa, which was obtained for the pure crosslinked Poly-TPGDA, an increase in the LC concentration provoked a sharp decrease in the moduli. A cross-over phenomenon was observed around 20 wt.% LC, indicating the lower moduli for EB-cured films with a higher LC concentration.

The inset of Figure 1 shows the stress-strain dependencies. The stress rise of EB-cured films is steep at low draw ratios, indicating the high modulus, whereas for UV-cured samples, a reduction in the modulus was observed. Increasing the LC concentration leads to a strong decrease in the modulus and the tensile strength of the films, accompanied with an increase in the elongation at break. Interestingly, upon addition of only 10 wt.% of LC, the Young modulus drops sharply, primarily due to the decrease in the cross-linking density of the polymer network. The stress–elongation curves of UV-cured films show yield stress, which means a higher viscoelasticity when compared with EB-cured samples. As a consequence, elongation at break in the UV-case becomes particularly important for high LC concentrations.

### 3.3. Dynamic Mechanical Measurements

The addition of LC to a polymeric material induces considerable changes in the mechanical behavior of the latter [10,11]. The cross-linking density of a polymer network decreases when the precursor mixture is diluted with LC, and the molecular weight of the strands between the cross-links increase, which yields a more flexible network with a lower plateau modulus [39].

**Figure 2:**
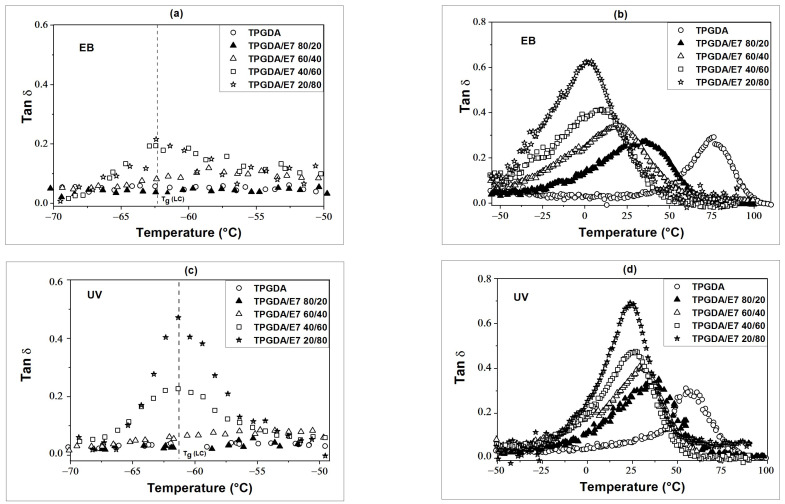
*tan δ* as function of temperature around *T_g_* (E7) (**a**,**c**) and *T_g_* poly (TPGDA) (**b**,**d**) at different LC concentrations for EB- and UV-cured films, respectively.

Figure 2 represents the evolution of *tan δ* around the glass transition temperatures (*T_g_*) of LC (*T*_g_ (LC)) and Poly-TPGDA (*T*_g_ (polymer)) as a function of temperature and composition for EB- and UV-cured films. *T_g_* values were deduced from the peak maxima of *tan δ*. In both the EB and UV cases, for less than 30 wt.% of LC only a single (*T*_g_ (polymer)) is observed since LC molecules are homogeneously dispersed in the polymer. In the range from 0 to 30 wt.% of E7, (*T*_g_ (polymer)) decreases from 55 °C to 29 °C for UV-cured and from 76 °C to 28 °C for EB-cured films, respectively, thus confirming the stronger plasticizing effect in the latter case. Beyond 30 wt.% of LC, the two compositions dependent *T*_g_’s appear (*T*_g_ (polymer)) and (*T*_g_ (LC)), thus indicating a phase separation between the LC and the polymer. In the vicinity of (*T_g_* (LC)) (Figure 2a,c), *tan δ* increases abruptly beyond 60 wt.% of E7, and in particular for UV-cured systems, reaching *tan δ* = 0.45 for 20 wt.% of Poly-TPGDA/80 wt.% of E7, and *tan δ* = 0.22 for the corresponding EB-cured blend. These results indicate stronger phase separation effects for UV-cured films. Moreover, EB-cured samples exhibit relatively wide *tan δ* peaks around (*T*_g_ (polymer)) and less regular and low intensity peaks around (*T*_g_ (LC)) (Figure 2a). This can be explained by the dispersion of a certain amount of LC molecules in the EB-cured polymer, providing more network elasticity. Around (*T_g_* (polymer)) (Figure 2b,d), peak maxima temperatures decrease with the LC concentration, accompanied with a sharp increase in peak intensities beyond 40 wt.% of E7. In the case of UV-cured films, (*T_g_* (polymer)) remains almost constant, whereas for EB-cured samples, it decreases and approaches (*T_g_* (LC)), indicating a certain LC/polymer miscibility of these blends.

**Figure 3:**
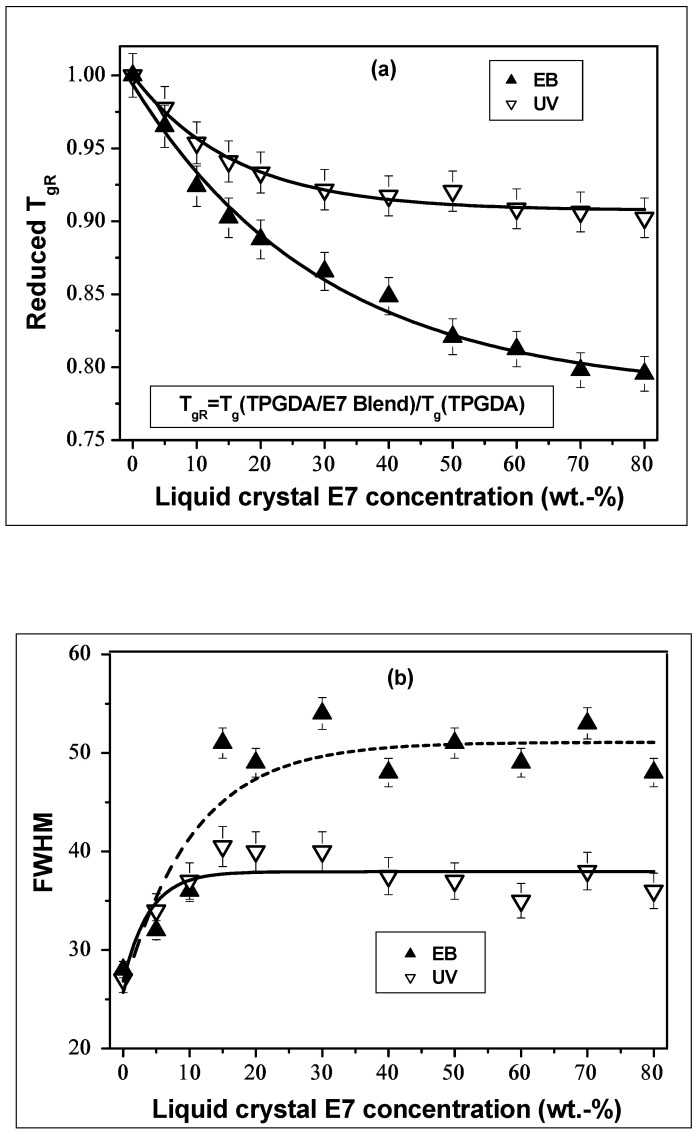
(**a**) Reduced *T_gR_*, and (**b**) Full Width at Half Maximum (FWHM) of *tan δ* around *(T_g_* (polymer)) as functions of LC concentration for both EB- and UV- cured TPGDA/E7.

Figure 3a represents the LC concentration dependence of the reduced *T*_gR_, defined as a ratio of *T*_g_ of cured TPGDA/E7 films to that of crosslinked TPGDA. Above 30 wt.% of LC, a plateau of *T_gR_* is observed for UV-cured samples, whereas for EB-cured films, the *T_gR_* curve continues to decrease, thus indicating a higher miscibility between the polymer and the LC when compared to the UV-cured samples. The Full Width at Half Maximum (FWHM) of *tan δ* peaks, given in Figure 3b, increases from 0 to 20 wt.% of E7. Plateau values were obtained at higher LC concentration, with a greater amplitude, thus confirming a better elasticity of the EB-cured polymer network.

**Figure 4:**
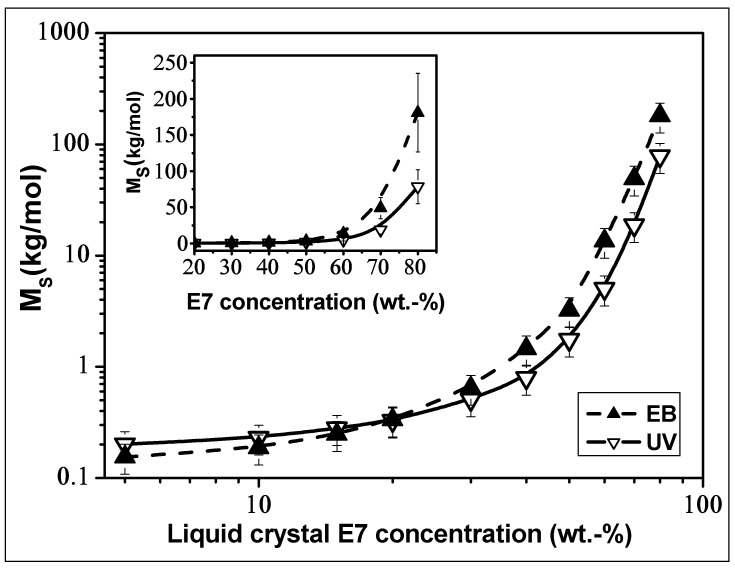
Molecular weight of strands (*M_s_*) between two adjacent cross-linking points of EB- and UV-cured TPGDA/E7 blends, as function of LC concentrations on a double logarithmic scaling. The inset shows the same results when applying a linear scaling.

Figure 4 describes the evolution of the average molar mass of strands between adjacent crosslinking points (*M_s_*), as a function of the LC concentrations for both UV- and EB-cured samples. The corresponding *M_s_* values were determined from the classical law of rubber elasticity [40]:

(1) $$E^{\prime }=3\rho RT/M_{s}$$

where *E_P_*’ represents the storage modulus at *T* = 100 °C, corresponding to the rubbery state plateau moduli, *ρ* is the volumetric density of the networks (determined in this report by weight measurements of the crosslinked samples with defined geometries, such as disks and parallelepipeds), and *R* stands for the constant of perfect gases. Figure 4 clearly shows that below 20 wt.% of LC, *M_s_* is slightly greater for UV-cured films indicating a higher crosslinking degree of EB-cured samples. Above 20 wt.% of LC, one observes inversion of the curves, i.e., the magnitudes of *M_s_* of EB-cured films become larger than those of UV-cured samples. Beyond 50 wt.% of LC, *M_s_* increases exponentially for both cases, and at 80 wt.% of LC, *M_s_* (EB-film) ≈ 3 × *M_s_* (UV-film), indicating a strong decrease in the crosslinking degree of the cured EB sample.

**Figure 5:**
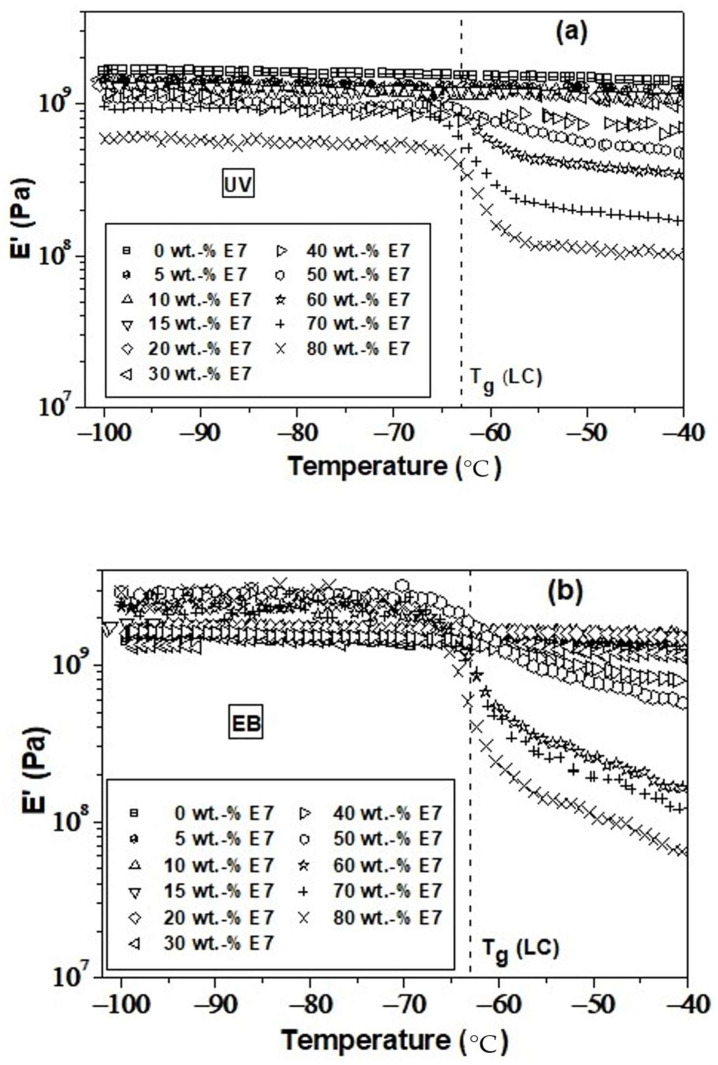
A detailed view of the storage tensile moduli versus temperature in the range from *T* = −100 °C to *T* = −40 °C. (**a**,**b**) displays results from EB- and UV-cured TPGDA/E7 films, respectively. The vertical dashed lines represent *T_g_* of the pure LC.

Figure 5 represents a detailed view of the storage tensile moduli versus temperature in the range from *T* = −100 °C to *T* = −40 °C. Figure 5a,b displays results from EB- and UV-cured films, respectively. In the glassy region of the polymer/LC systems, at temperatures below −70 °C, plateau values of the storage tensile moduli were observed for both UV- and EB-irradiated samples, which strongly depend on the LC concentrations.

For UV-cured films (Figure 5a), with exception of 60 wt.% of LC, increasing LC content leads to a decrease in the plateau moduli from 1.6 GPa for pure polymerized TPGDA to 0.6 GPa for the blend containing 80 wt.% of LC. The plateau moduli of samples possessing between 5 and 70 wt.% of LC can be found roughly in the same order of magnitude, whereas the modulus of the 80 wt.% of LC film was found much below the values of the other samples. It should be mentioned that films exhibiting LC concentrations below 40 wt.% do not show any discontinuity around (*T*_g_ (LC)). Note the increasing drop of moduli at (*T*_g_ (LC)) for samples exceeding 40 wt.% of LC. Interestingly, the highest drop of 0.5 GPa was found for 80 wt.% of LC films.

For EB-cured films (Figure 5b), between 0 and 40 wt.% of LC, the corresponding plateau values roughly overlap and no clear dependence on the LC content was observed. For LC concentrations higher than 40 wt.%, the EB-curves show widely dispersed plateau moduli of up to 1 GPa, higher than those of films with a lower LC content. This surprising behavior was not observed for UV-cured samples.

**Figure 6:**
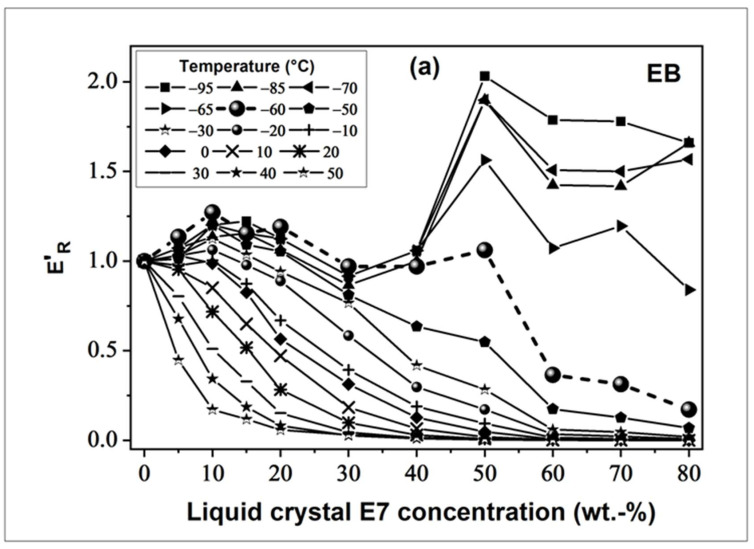

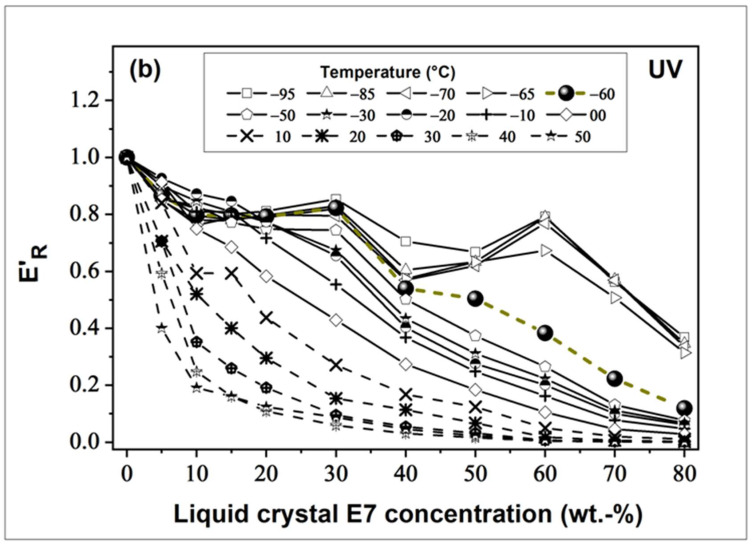
Reduced storage tensile moduli *E’_R_* as function of LC concentration determined at different temperatures for (**a**) EB- and (**b**) UV- cured TPGDA/E7 films.

To achieve a better understanding of the observed phenomena, reduced storage moduli (*E’_R_*) defined by *E’_R_* = *E’*(cured TPGDA/LC)/*E’*(cured TPGDA) are shown in Figure 6a,b as a function of the LC concentration in the range from −95 °C to 60 °C for EB- and UV-cured samples, respectively. Generally, low *E’_R_* values were obtained at high temperatures, and vice-versa. The isotherm corresponding to *T_g_* (pure LC) divides the graphs into two distinct parts.

For temperatures above *T* = −60 °C, *E’_R_* begins to decrease and drops sharply towards high temperatures. This behavior can be related to the sorption of the LC as a penetrant within the polymer matrix and has been described in the literature as a plasticizing effect. A strong loss of rigidity is observed with increasing LC concentration, confirmed by a strong drop of *T*_g_ (see Figure 3a).

Furthermore, polymer network formations depends on the LC concentration in the reactive initial blends prior to curing, for example, the network developed with 70 wt.% of LC exhibits a much greater distance between two adjacent cross-linking points than the network prepared with 30 wt.% of LC. As mentioned before, an increasing *M*_s_ value leads to an enhanced network elasticity while the rubbery state plateau moduli *E_P_’* (see Equation (1)) will be reduced.

For EB-cured films (Figure 6a), below *T* = −60 °C and for a LC content higher than 30 wt.%, *E’_R_* increases sharply, reaching twice its initial value (*E’_R_* = 2.03) at 50 wt.% of LC, and stabilizes around *E’_R_* = 1.6 for high LC concentrations. The composition dependent appearance of sub-micron-sized phase separated LC domains within the polymer matrix, at temperatures below (*T*_g_ (LC)), can explain these observations. These glassy LC domains lead to a reinforced structure of the polymer/LC system and possess an improved mechanical behavior when compared to the virgin polymer. It has already been demonstrated in the literature that parameters, such as dimension, volumetric, and spatial densities of filler particles strongly influence the modulus [41].

In contrast to the results obtained from EB-cured films, no enhancement effect on *E’_R_* was detected for UV-cured TPGDA/LC samples (Figure 6b), when compared to pure cured TPGDA, even at the lowest temperature measured (*T* = −95 °C). At this temperature, *E’_R_* drops from 0.85 (30 wt.% of LC) to 0.67 (50 wt.% of LC), then rises to 0.79 (60 wt.% of LC), and suddenly relapses for higher LC concentrations. *E’_R_* decreases with an increasing LC content in the temperature range from *T* = 25 °C to −15 °C due to the plasticizing effect induced by the presence of the dispersed LC molecules in the polymer matrix. Only in the case of polymer/LC blends with more than 30 wt.% of LC and for temperatures lower than −15 °C, a small reinforcement effect was seen, leading to a change in the shape of the decreasing *E’_R_* curves. As for the EB-case, the change in the shape of the curves can be explained by the appearance of sub-micron-sized glassy LC domains, which stabilize the modulus. These results can be related to the different irradiation methods and their consequences on the sample morphology and the polymer network structure.

**Figure 7:**
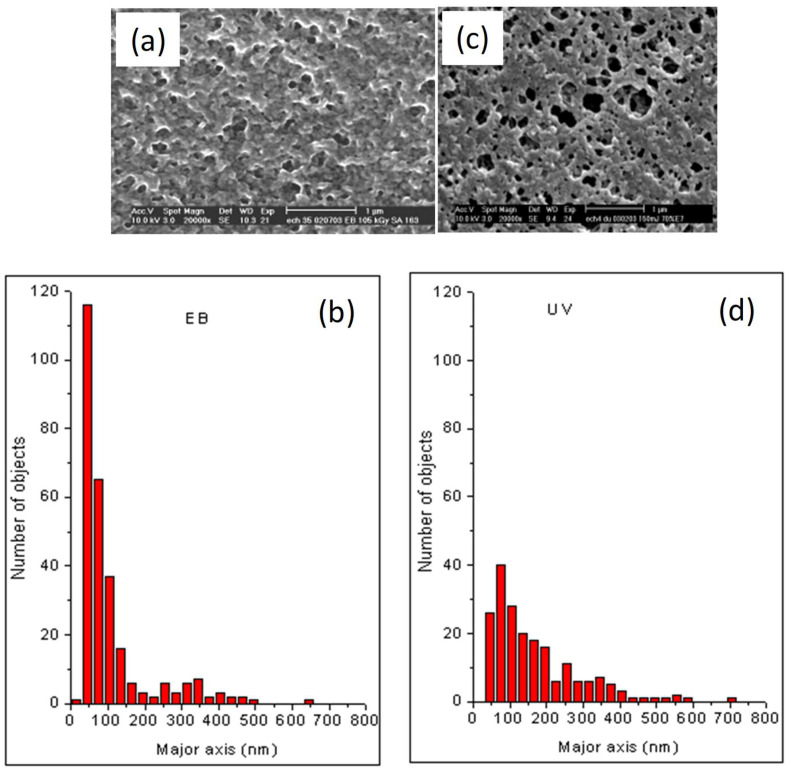
(**a**) A representative micrograph obtained from SEM observations is presented, showing the morphology of an EB-cured 30 wt.% of TPGDA/70 wt.% of E7 film. (**b**) Histogram obtained by image processing of the SEM picture from Figure 7a, taking into account the size distribution of the LC domains, and assuming the presence of ellipsoids characterized by a major and a minor axis. The corresponding SEM analysis of a UV-cured 30 wt.% of TPGDA/70 wt.% of E7 film is given in (**c**,**d**). In both cases, the same experimental conditions were used for the morphological observations of the samples.

Representative SEM micrographs of EB- and UV-cured 30 wt.% of TPGDA/70 wt.% of E7 films are exhibited in Figure 7, applying the same magnification. These pictures show a large number of dark domains, corresponding to empty holes after careful LC removal embedded in a brighter continuous environment that represents the polymer matrix. LC domains are randomly distributed over the films and no preferential orientation of these domains was observed. Image processing of the SEM images was undertaken using freely available software from Scion. The results obtained from this granulometric data treatment were compared with a standard manual method by tracing the encirclements of phase separated LC domains on semi-transparent paper, which was then scanned and analyzed by Scion software.

Figure 7a represents a typical image of the morphology of an EB-cured 70 wt.% of E7/30 wt.% of TPGDA film, revealing a large number of segregated nano-sized LC domains, exhibiting a maximum of their major axis around 50 nm (see histogram on Figure 7b). These LC domains present a nearly monodisperse distribution of sizes and forms and can act as reinforcing filler particles below (*T*_g_ (LC)) which is in good agreement with the observed mechanical enhancement effect.

On the other hand, Figure 7c shows the morphology of the same monomer/LC mixture irradiated by UV light, revealing a smaller number of more irregular shaped larger LC domains when compared to the EB-cured sample. The maximum of the major axis of the LC domain size distribution of the UV-cured film was found around 80 nm, together with a relatively long tailing up to 700 nm (see histogram on Figure 7d). Moreover, these irregularly shaped LC domains are often interconnected (revealed by strongly magnified SEM pictures, not shown here), forming larger objects, also explaining the absence of a mechanical reinforcing effect.

Furthermore, the presence of polymer network heterogeneities should be mentioned due to the free radical polymerization/crosslinking processes. As a consequence, regions with highly and loosely cross-linked polymer networks may be created locally. These heterogeneities can be formed, especially in the case of the UV-cured sample, due to the initiation step and may contribute to the absence of the mechanical reinforcement effect. Indeed, during the initiation step of the photo-polymerization process, the reaction starts first around the photoinitiator radicals, forming polymer network spots, which might be different from one spot to another, depending on the local solubility of the initiator in the monomer. As a consequence, this might induce some heterogeneity, not only in the obtained polymer networks, but also in the morphologies of the phase separated polymer/LC system. However, under EB exposure; the electrons directly react with the double bonds of TPGDA, which should be homogenous in the mixture, and as a result, homogenous networks and morphologies are likely to be formed throughout the EB-cured polymer/LC film.

## 4. Conclusions

In this report, static and dynamic mechanical studies of UV- and EB-cured Tripropyleneglycoldiacrylate were presented. An important decrease in the mechanical strength of the polymeric films was observed when the polymer precursor blend contains E7 as a model for a low molar weight nematic LC. Below 30 wt.% of E7, LC molecules are well dispersed in the polymer matrix thus weakening its mechanical properties by a plasticizing effect. Phase separated structures were obtained above 30 wt.% of LC, where E7 domains were formed in the polymer networks. In this concentration range, at temperatures below the LC glass transition, EB-cured TPGDA/LC films present strong and unusual enhancements of their mechanical modulus. Therefore, tunable mechanical properties can be obtained from such phase separated polymer/LC materials, depending on the LC glass transition temperatures and sample compositions. This reinforcement effect can be explained by the sample morphology, showing a large number of nano-sized monodisperse LC domains in the EB-cured films. On the other hand, UV-cured samples did not show a reinforcement effect due to the large size distribution of irregularly shaped LC droplets.

## Data Availability

Data set presented in this study is available in this article.
